# A Study of the Ionic Liquid-Based Ultrasonic-Assisted Extraction of Isoliquiritigenin from *Glycyrrhiza uralensis*

**DOI:** 10.1155/2020/7102046

**Published:** 2020-10-01

**Authors:** Jingwei Hao, Jiahui Liu, Lei Zhang, Yunrong Jing, Yubin Ji

**Affiliations:** ^1^Center for Life Science and Environmental Science, Harbin University of Commerce, Harbin 150076, China; ^2^College of Life Sciences and Technology, Mudanjiang Normal University, Mudanjiang 157011, China

## Abstract

We successfully extracted isoliquiritigenin from *Glycyrrhiza uralensis* through the utilization of an ionic liquid-based ultrasonic-assisted extraction (ILUAE) approach. Briefly, we utilized the solution of 1-butyl-3-methylimidazolium bromide ([BMIM]Br) as solvent and optimized key ILUAE parameters such as solid-liquid ratios, concentrations of ionic liquids, and the times of ultrasonication. Based on a single-factor experiment, we utilized the response surface method (RSM) approach to optimize the extraction procedure. The approach revealed that the optimal energy consumption time was 120 min, with the ultrasonic extraction temperature of 60°C. Using these optimized parameters together with the solid-liquid ratio (dried *G. uralensis* powder: [BMIM]Br of 0.3 mol/L) of 1 : 16.163 and the [BMIM]Br of 0.3 mol/L, we achieved a 0.665 mg/g extraction yield. Overall, these findings thus indicate that we were able to effectively use ILUAE as an efficient approach to reliably extract isoliquiritigenin in a reproducible and environmentally friendly manner.

## 1. Introduction

*Glycyrrhiza uralensis* Fisch (sweet root, sweet grace, or Ural licorice) is a plant that is frequently used for traditional medicinal purposes in China [1–3]. Isoliquiritigenin is a flavonoid that can be extracted from the roots of *G. uralensis*, and that has been found to exhibit diverse pharmacological effects following its isolation from *Glycyrrhiza* species [4–8]. Indeed, isoliquiritigenin has been shown to be capable of inhibiting monoamine oxidase [4]. Strikingly, it has also been shown to suppress tumor cell proliferation and to promote apoptotic tumor cell death, exhibiting therapeutic efficacy 20-fold greater than that observed for any other extracts from *Glycyrrhiza* when used to treat human breast cancer cells [5]. Owing to these promising properties, isoliquiritigenin has attracted substantial interest as a potential pharmaceutical agent. However, its relatively low abundance in *G. uralensis* and its poor solubility have made its extraction for pharmacological research challenging [9–13].

Ionic liquids are nonmolecular solvents that exhibit limited volubility, good stability, low melting point, and ability to readily dissolve certain compounds [14–18]. As such, these liquids are highly flexible and have commonly been employed in traditional Chinese medicine (TCM) as a means of reducing extraction times, improving extraction efficiency rates, and reducing the negative environmental impact associated with traditional solvents [19]. As these ionic solvents can fully penetrate medicinal raw materials, they can effectively extract the compounds of interest located therein, resulting in the observed increases in extraction rates [20–27].

Conventionally, isoliquiritigenin is extracted from *G. uralensis* via heating, boiling, or refluxing solutions containing plant materials and ethanol. However, these methods are inefficient and result in isoliquiritigenin loss, poor extraction rates, and the consumption of large organic solvent volumes.

Recently, studies have demonstrated the value of leveraging the thermal, mechanical, and cavitation properties of ultrasonication as a means of improving compound extraction via accelerating dissolution, release, and diffusion of compounds employed for TCM purposes [28–30]. Such ultrasonic extraction protocols can simultaneously decrease extraction time while increasing extraction rates, thus making them ideal for isolating low-abundance compounds. These approaches can also function at low temperatures and can be applied in a variety of contexts [31]. Herein, we extracted isoliquiritigenin from *G. uralensis* via an optimized approach using ionic liquids [32].

## 2. Materials and Methods

### 2.1. Reagents

*G. uralensis* was from Mudanjiang Pharmaceutical Chain Co. Ltd. while the isoliquiritigenin standard (98% pure) was from the National Institute for the Control of Pharmaceutical and Biological Products (Beijing, China). All the ILs, namely, 1-butyl-3-methylimidazolium bromide ([BMIM]Br), 1-butyl-3-methylimidazolium chlorinate ([BMIM]Cl), 1-butyl-3-methylimidazolium hydrogen sulfate ([BMIM][HSO4]), and 1-butyl-3-methylimidazolium nitrate ([BMIM][NO3]), were from Shanghai Aichun Biotechnology Co. Ltd. (Shanghai, China). Anhydrous ethanol was from Nanjing Xingsha Chemical Co. Ltd. while methanol was from Puyang Wangda Chemical Co. Ltd. (Nanjing, China), and acetonitrile was from Jinan Century Tongda Chemical Co. Ltd. (Jinan, China). All other analytical-grade chemicals and solvents were from Beijing Chemical Reagents Co. (Beijing, China).

### 2.2. Analytical Conditions

For this study, we utilized a Kq-400 db ultrasonic cleaner from Shenzhen Keweida Ultrasonic Equipment Co. Ltd. (Shenzhen, China), a 98-1-b electronic temperature-regulating heating sleeve from Heze Shengbang Instrument Development Co. Ltd. (Heze, China), a tp-213 electronic balance from Shanghai Ziyi Instrument Equipment Co. Ltd. (Shanghai, China), and a Waters 2695 HPLC instrument from Waters Co. (Milford, MA, USA). In addition, a HiQ Sil-C18 reversed-phase column (4.6 mm × 250 mm, 5 *μ*m, KYA TECH Corp., Tokyo, Japan) was used for chromatographic separation.

**Figure 1:**
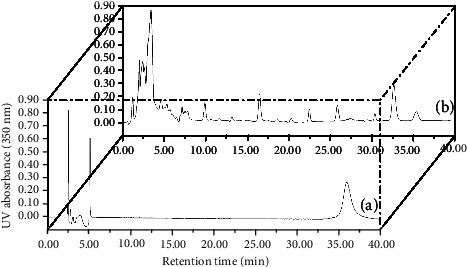
HPLC chromatogram for isoliquiritigenin in standard solutions (a) and sample extract (b).

We utilized an acetonitrile-water-acetic acid (32 : 68 : 0.5, v/v/v) mobile phase for HPLC analyses, with a 1.0 mL/min flow rate, a 10 *μ*L injection volume, and a column temperature at 25°C column temperature. Isoliquiritigenin was then detected based on absorbance at 350 nm (see Figure 1).

The resultant calibration curve used the formula (*Y* = 3 × 10^7^) (*X* + 2 × 10^6^) (*R*^2^ = 0.9911, *X*: isoliquiritigenin concentration, *Y*: peak area), indicating a good linear fit for isoliquiritigenin.

### 2.3. Glycyrrhiza Water Content Assessment

*G. uralensis* was ground to produce a fine powder, with three 1 g portions of this powder then being weighed and transferred to an oven for storage for 24 h, after which the absolute dry weight was calculated and used to determine moisture contents. Ultimately, *Glycyrrhiza uralensis* was found to have a 3.4% water content:

(1) $$\begin{array}{c} Y=\frac{\left(a-b\right)}{a}\times 100\%. \end{array}$$

Where *Y* corresponds to water content, as a percentage, *a* represents wet weight and *b* represents dry weight.

### 2.4. Optimization of Isoliquiritigenin Extraction Using a Factorial Design

The univariate method was used to optimize the following parameters: the kinds of ionic liquids, ionic liquid concentration, solid liquid ratio (g/mL), temperature (°C), and ultrasonic time (min).

#### 2.4.1. Screening of Ionic Liquids

The anion identity is considered to strongly influence the ionic liquid's properties. We utilized [BMIM]Br, [BMIM]Cl, [BMIM][HSO_4_], and [BMIM][NO_3_] for these analyses. We utilized a Kq-400 db ultrasonic unit that had a 100 W maximum power. The following extraction parameters were utilized: 0.4 g of a dried sample was combined with 0.3 mol/L of the different kinds of IL used with a solid-liquid ratio of 1 : 15 and then extracted for 120 min by UAE at a temperature of 60°C. The results showed that the ionic liquids based on Br^−^ was the most efficient of the ionic liquids tested, and [BMIM]Br was selected for subsequent experiments.

#### 2.4.2. Effect of Concentration

The extractions were carried out in aqueous solutions with a range of [BMIM]Br IL concentrations of (0.05, 0.1, 0.2, 0.3, 0.4, 0.5, and 0.6 mol/L), 0.4 g of the dried sample was combined with different [BMIM]Br IL concentrations used with a solid-liquid ratio of 1 : 15, and then extracted for 120 min by UAE at a temperature of 60°C. When with the 0.3 mol/L [BMIM]Br, the extraction rate of isoliquiritigenin was the highest. Therefore, 0.3 mol/L [BMIM]Br was selected for subsequent experiments.

#### 2.4.3. Effect of the Solid-Liquid Ratios

The solid-liquid ratio is an important factor because large solvent volumes could make the procedure difficult and lead to unnecessary solvent waste. By contrast, small solvent volumes may lead to incomplete extraction. A series of experiments were carried out with different solid-liquid ratios (10, 15, 20, 25, 30, and 40 g/mL) The following extraction parameters were utilized: 0.4 g of a dried sample was combined with the 0.3 mol/L [BMIM]Br, the duration of ultrasonication was 120 min, the temperature was 60°C, and the ultrasonication power was 100 W. Thus, the solid-liquid ratio of 1 : 15 was used in subsequent experiments.

#### 2.4.4. Effect of the Temperatures

The temperature is important in determining the extraction yields. Extraction temperatures of 20°C, 30°C, 40°C, 50°C, 60°C, 70°C, and 80°C were investigated. The following extraction parameters were utilized: combined 0.4 g of a dried sample with the 0.3 mol/L [BMIM]Br used with a solid-liquid ratio of 1 : 15, the duration of ultrasonication was 120 min, and the ultrasonication power was 100 W. The result showed that the extraction rate was the highest when the extraction temperature was 60°C. Therefore, the extraction temperature of 60°C was selected for subsequent experiments.

#### 2.4.5. Effect of the Ultrasonication Time

Optimization of the ultrasonication time is very important to ensure efficient extraction. Ultrasonication times of 30, 60, 90, 120, 150, 180, and 210 min were investigated. 0.4 g of accurately weighed sample was extracted with 0.3 mol/L [BMIM]Br aqueous solutions and with a solid-liquid ratio of 1 : 15 at 60°C, respectively. When the extraction time was 90 min, the extraction rate of isoliquiritigenin reached the highest value.

### 2.5. Optimization Ultrasonic-Assisted Extraction Methods via the Response Surface Method (RSM)

The RSM approach was employed to understand the interaction between optimized conditions via the use of the Box-Behnken data-processing software. Based on a single-factor investigation approach, we selected the [BMIM]Br concentration, extraction time, and solid-liquid ratio as independent variables and the isoliquiritigenin extraction rate as the dependent variable. The ultrasonic extraction temperature was fixed at 60°C, with all other conditions then being optimized accordingly.

### 2.6. Assessment of ILUAE Stability and Repeatability

We evaluated the stability of this approach by dissolving isoliquiritigenin standards in [BMIM]Br via UAE using optimized assay conditions ([BMIM]Br = 0.3 mol/L; solid-liquid ratio = 1 : 16; ultrasonic time = 120 min; 60°C; and 100 W). Isoliquiritigenin recovery was then evaluated. Reproducibility was assessed by processing five identically weighted samples (0.4 g) under optimized extraction conditions.

### 2.7. Reference and Conventional Extraction Approaches

Two different comparisons were made to the ILUAE extraction method. In one approach, a 75% ethanol aqueous solution was used as a reference solvent for isoliquiritigenin UAE from *G. uralensis*. Extraction was additionally conducted under optimized extraction conditions, with solvent type being altered. Additionally, a 75% ethanol aqueous solution was used for the reflux extraction of isoliquiritigenin with 1 : 25 solid-liquid ratio, and 80°C extraction temperature, and 1.5 h extraction duration. Then, filtrates were collected and volumes were assessed to determine extraction rates. We combined a 0.4 g dried sample with 10 mL of 75% aqueous ethanol and performed extraction for 90 min at 100 W ultrasonic power.

### 2.8. Statistical Analysis

To indicate the extraction efficiency of isoliquiritigenin, one-way ANOVA and Duncan's multiple range test was used to determine the significant differences between experiments with different conditions. All statistical significance was accepted when *α* < 0.05. The results of the HPLC analysis were expressed as means of extraction efficiency ± SD. Data analyses were conducted in SPSS 22.0 software.

## 3. Results and Discussion

### 3.1. Single-Factor Experimental Design

#### 3.1.1. An Evaluation of the Impact of Different IL Solutions on UAE-Mediated Isoliquiritigenin Extraction from *G. uralensis*

**Figure 2:**
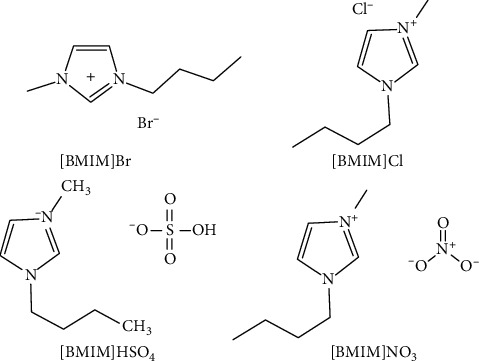
Chemical structures for ILs.

**Figure 3:**
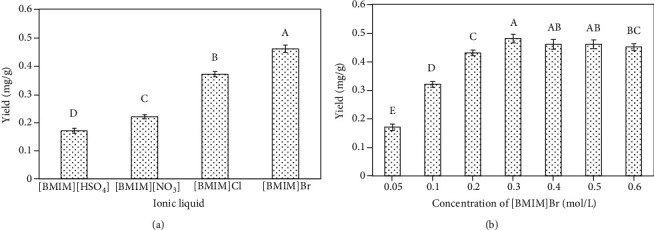
(a) The impact of IL type. The following extraction parameters were utilized: 0.4 g of dried sample combined with an IL concentration of 0.3 mol/L was used with a solid-liquid ratio of 1 : 15, a 120 min ultrasonication duration, a temperature of 60°C, and a 100 W ultrasonic power. (b) The impact of IL concentrations. The following extraction parameters were utilized: 0.4 g of dried sample combined with [BMIM]Br aqueous solution was used with a solid-liquid ratio of 1 : 15, a 120 min ultrasonication duration, a temperature of 60°C, and a 100 W ultrasonic power. The different uppercase letters on the bars represent significant differences (*P* < 0.05) between treatments.

The specific composition of a given IL ultimately determines its physical properties. For this study, we selected four different IL solutions containing different anions ([BMIM]Br, [BMIM]Cl, [BMIM][HSO_4_], and [BMIM][NO_3_]; see Figure 2). Anion identity can profoundly impact the properties of these IL solutions [32]. All of these selected IL solutions were miscible with water at all concentrations. Our analyses revealed that Br^−^ was a more efficient mediator of isoliquiritigenin extraction than any other tested solutions (*P* < 0.05). Indeed, when [BMIM]Br was added, higher isoliquiritigenin extraction rates were obtained (see Figure 3(a)). As such, [BMIM]Br was used in downstream experiments.

#### 3.1.2. The Impact of IL Concentration on Isoliquiritigenin Extraction

We next formulated ILs at eight different concentrations (0.05, 0.1, 0.2, 0.3, 0.4, 0.5, and 0.6 mol/L) to assess the impact of such concentrations on extraction efficiency. Additional extraction conditions were as follows: 0.4 g of dried sample was combined with different concentrations of ionic liquid aqueous solution at the solid-liquid ratio of 1 : 15, ultrasonication temperature of 60°C, and 1.5 h ultrasonication duration. A clear dose-dependent impact of IL concentration on isoliquiritigenin yield was observed via this approach (see Figure 2(b)), with [BMIM]Br concentrations of 0.05-0.3 mol/L being associated with rising isoliquiritigenin extraction rates that decreased significantly at higher concentrations. The results showed that there were significant differences among different concentrations (*P* < 0.05). Given that peak extraction was achieved at an IL concentration of 0.3 mol/L, this concentration was utilized in downstream experiments. The observed dose-dependent relationship between IL concentration and extraction efficiency may suggest that higher-viscosity IL solutions may result in poorer penetration of the raw plant materials.

#### 3.1.3. The Impact of Solid-Liquid Ratios on Isoliquiritigenin Extraction

The ratio of raw plant material to solvent can be a key determinant of extraction efficiency. Specifically, higher solvent volumes generally result in enhanced extraction efficiency, but excessively large volumes can result in complex extractions and substantial unnecessary waste.

**Figure 4:**
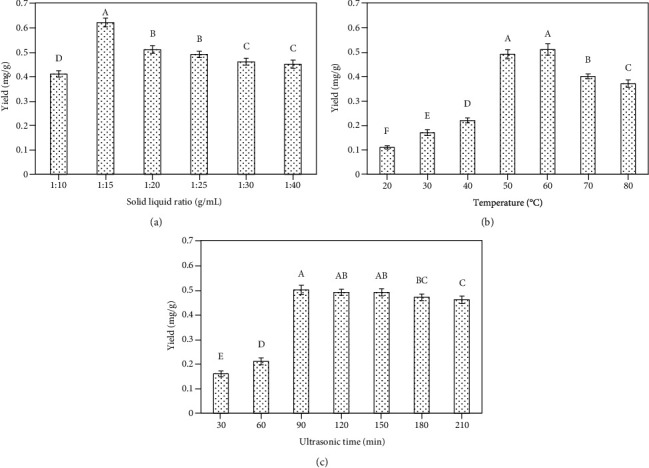
Extraction condition optimization. The following extraction parameters were utilized: (a) 0.4 g of a dried sample was combined with the [BMIM]Br concentration of 0.3 mol/L, the duration of ultrasonication was 120 min, the temperature was 60°C, and the ultrasonication power was 100 W. (b) 0.4 g of a dried sample was combined with the [BMIM]Br concentration of 0.3 mol/L, the duration of ultrasonication was 120 min, the temperature was 60°C, and the ultrasonication power was 100 W. The solid-1iquid ratio for this extraction was 1 : 15. (c) 0.4 g of a dried sample was combined with the [BMIM]Br concentration of 0.3 mol/L, the duration of ultrasonication was 30-210 min, the temperature was 60°C, the solid-liquid ratio was 1 : 15, and the ultrasonication power was 100 W. The different uppercase letters on the bars represent significant differences (*P* <0.05) between treatments.

We observed significant increases in isoliquiritigenin extraction rates when the solid-liquid ratios were between 1 : 10 and 1 : 15 (*P* < 0.05) (see Figure 4(a)), potentially suggesting that at ratios below 1 : 10, the viscosity of the extract was too great, thereby preventing efficient isoliquiritigenin extraction. Increasing the solid-liquid ratio can improve isoliquiritigenin extraction such that peak extraction was achieved at a ratio of 1 : 15. As such, subsequent single-factor experiments were conducted using this 1 : 15 solid-liquid ratio.

#### 3.1.4. The Impact of Different Temperatures on Isoliquiritigenin Extraction Efficiency

Temperature was found to be a key factor associated with extraction efficiency, with this efficiency rising gradually as temperature increased. There were significant differences between different temperatures (*P* < 0.05). This increase ceased at temperatures above 60°C, after which isoliquiritigenin extraction efficiency decreased. This result may be attributable to temperature-dependent increases in extraction efficiency that are disrupted when the temperature rises too high and begins to breakdown the structure of isoliquiritigenin, resulting in an apparent decrease in extraction rate (see Figure 4(b)).

#### 3.1.5. The Impact of Ultrasonication Time on Extraction Efficiency

Next, extraction was conducted across a range of extraction times (30, 60, 90, 120, 150, 180, and 210 min). There were significant differences between different ultrasonication times (*P* <0.05). At extraction times < 1 h, we observed very low isoliquiritigenin extraction rates, likely because this timeframe did not offer sufficient time for the solvent to penetrate into the root of *G. uralensis* (see Figure 4(c)). At extraction times greater than 1.5 h, extraction rates also tended to decrease, suggesting that prolonged extraction may result in the destruction of flavonoids such as isoliquiritigenin, leading to a lower extraction rate. Peak isoliquiritigenin extraction was thus observed after 1.5 h, and as such this time was used for subsequent experiments.

### 3.2. Response Surface Methodology-Mediated Parameter Optimization

After evaluating these extraction-related parameters individually, we next explored the interactions between these factors via RSM in an effort to better optimize IL concentrations, solid-liquid ratios, and extraction time values. For this analysis, these three parameters were treated as independent variables, and the isoliquiritigenin extraction index served as dependent variable.

Experimental randomization was conducted as detailed in Table 1 in an effort to maximize the impact of unexplained variability on the extraction rates. In total, we conducted 17 tests, with 5 replicates (runs 1, 2, 10, 11, and 15; see Table 1) being used to estimate the pure error sum of squares.

The predicted *R*^2^ value of 0.8758 was reasonably consistent with the adjusted *R*^2^ value of 0.9541, and our precision ratio was 21.783, indicating adequate precision (see Table 2). Furthermore, our model had very high *F* values and low *P* values (*P* < 0.0001) for two responses. The *F* value of 37.95 suggests that there is only a 0.01% chance that this value is the result of noise. Indeed, any “Prob > *F*” values of <0.0500 are significant, whereas values > 0.1 are not. Based on these criteria, the following terms in our model were significant: A, B, C, AB, A^2^, B^2^, and C^2^ (see Table 3).

The predicted *R*^2^ value of 0.8758 was reasonably consistent with the adjusted *R*^2^ value of 0.9541, with the difference being less than 0.2. The signal-to-noise ratio was measured by the “Adeq Precision” metric in the predictive software. As our ratio was 21.783, which was greater than the minimum desirable level of 4, this suggested that our signal was adequate and this model could be reliably utilized to navigate the design space.

The results of this analysis revealed that these three independent variables were related to one another based upon the second-order polynomial equation: yield (mg/g) = 0.54 + 0.078A + 0.07B + 0.058C + 0.032AB + 0.012 AC − 0.0025 BC − 0.03A^2^ − 0.035B^2^ − 0.14C^2^.

**Figure 5:**
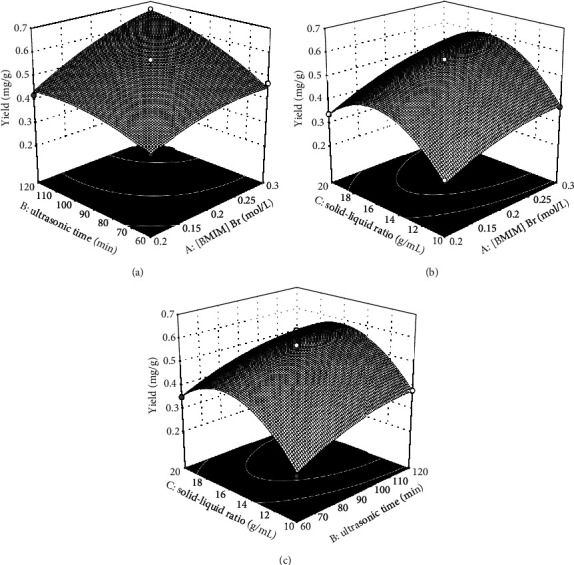
Response surface plots revealing the impact of the indicated variables on analyte extraction rates: (a) interactions between [BMIM]Br concentrations and duration of ultrasonication; (b) interactions between [BMIM]Br concentrations and solid-liquid ratio values; (c) interaction between ultrasonication duration and solid-liquid ratio.

Response surfaces corresponding to the impact of the indicated independent variables on average isoliquiritigenin extraction efficiency are shown in Figure 5, with the interactions between [BMIM]Br and solid-liquid ratio, between [BMIM]Br and ultrasonication time, and between solid-liquid ratio and ultrasonication time shown in Figures 5(a), 5(b), and 5(c), respectively. Based on these analyses, the predictive software estimated that with a [BMIM]Br concentration of 0.3 mol/L, and the solid-liquid ratio of 1 : 16.163, and the ultrasonication time of 120 min, yield could be as high as 0.665 mg/g.

### 3.3. Verification Tests

Verification testing was conducted thrice under pint prediction RSM conditions ([BMIM]Br = 0.3 mol/L, solid-liquid ratio = 1 : 16.163, ultrasonication time = 120 min). These analyses yielded an actual extraction efficiency of 0.651 ± 0.014 mg/g.

### 3.4. Assessment of ILUAE Stability and Repeatability

Isoliquiritigenin standard stability (10.0 and 20.0 *μ*g/mL) in 0.3 mol/L [BMIM]Br, with a solid-liquid ratio of 1 : 16, a temperature of 60°C, an ultrasonication duration of 120 min, and 100 W power, was assessed. Average recovery rates were between 100.2% and 102.3%, and no changes in retention times were observed, suggesting that this compound remains stable in a [BMIM]Br solution.

### 3.5. Comparison of Different Extraction Methods

We utilized two different approaches to compare the relative strengths of our ILUAE extraction approach. The extraction process was as follows: 0.4 g of dried samples was combined with the 75% ethanol aqueous solution, after which the solution was refluxed for 90 min. For this approach, the yield was 0.17 ± 0.005 mg/g. The second extraction approach was identical to the strategy, but with ultrasonic extraction instead being utilized. The ultrasonication approach disrupts the external structure of raw materials at certain frequencies, enabling the solvent to better penetrate the material. The results had better extraction rates, as well as more rapid extraction. In the present study, the extraction rate was 0.28 ± 0.009 mg/g.

## 4. Conclusions

Herein, we utilized a [BMIM]Br-based UAE strategy to explore the optimal conditions for extracting isoliquiritigenin from *G. uralensis* through the use of a Box-Behnken design. Ionic liquids are promising tools for extracting active compounds from raw medicinal materials. An ultrasonication-based extraction method was selected owing to its ability to disrupt the external structure of raw medicinal materials, facilitating enhanced solvent penetration, more rapid extraction, and a higher extraction rate. The approach can be further optimized by tuning the properties of the ionic liquids used therein. Herein, we successfully developed an ILUAE method to reliably extract isoliquiritigenin from *G. uralensis*. Briefly, we utilized a Kq-400 db ultrasonic unit (100 W maximum power), and combined 0.4 g of dried sample with [BMIM]Br aqueous solution. Then, we optimized IL concentrations, solid-liquid ratios, temperatures, and extraction times in order to achieve maximal extraction efficiency. To better explore how these factors were associated with one another, we utilized an RSM approach with the Box-Behnken software to further optimize these extraction procedures. For the analysis, ionic liquid concentrations, extraction times, and solid-liquid ratios were used as independent variables, while the rate of isoliquiritigenin was the dependent variable. Through the approach, we determined that the optimal conditions were as follows: [BMIM]Br = 0.3 mol/L, solid-liquid ratio = 1 : 16.163, and ultrasonication time = 120 min. Under these conditions, the maximum extraction efficiency of 0.665 mg/g could be achieved.

As a novel extraction method that combines an environmentally friendly ionic liquid with ultrasonic-assisted extraction technology, ILUAE technology not only is fast and efficient but also can be widely used for the extraction of bioactive components of TCM owing to its limited solvent and energy consumption rates. The ionic liquid-based ultrasonic-assisted extraction of isoliquiritigenin from *G. uralensis* required low amounts of energy and time, while also being able to conduct and being relatively stable. Compared with traditional reflux extraction methods that require sample heating, this approach yields high extraction efficiency while avoiding the deactivation of active compounds at high temperatures. It further overcomes other shortcomings of traditional extraction methods, such as carbonization, hydrolysis, significant organic solvent consumption, flammability, and explosion risk.

## Figures and Tables

**Table 1:** The Box-Behnken experimental design.

Run	Factor A Concentration of [BMIM]Br (mol/L)	Factor B Extraction time (min)	Factor C Solid-liquid ratio (g/mL)
1	0.2	90	1 : 15
2	0.2	90	1 : 15
3	0.2	120	1 : 10
4	0.3	90	1 : 10
5	0.1	60	1 : 15
6	0.2	60	1 : 10
7	0.3	90	1 : 20
8	0.1	120	1 : 15
9	0.2	60	1 : 20
10	0.2	90	1 : 15
11	0.2	90	1 : 15
12	0.3	60	1 : 15
13	0.2	120	1 : 20
14	0.1	90	1 : 10
15	0.2	90	1 : 15
16	0.1	90	1 : 20
17	0.3	120	1 : 15

**Table 2:** The credibility analysis of the regression equations.

Index mark^a^	Extraction efficiency of isoliquiritigenin
Std. dev.	0.025
Mean	0.44
C.V.%	5.74
PRESS	0.028
*R*-squared	0.9799
Adj *R*-squared	0.9541
Pred *R*-squared	0.8758
Adeq precision	21.783

**Table 3:** Test of significance for regression coefficient.

Source	Sum of squares	Df	Mean square	*F* value	*P* value
Model	0.22	9	0.025	37.95	<0.0001
A-[BMIM]Br	0.048	1	0.048	74.25	<0.0001
B-ultrasonic time	0.039	1	0.039	60.57	0.0001
C-solid-liquid ratio	0.026	1	0.026	40.87	0.0004
AB	4.225 × 10^−3^	1	4.225 × 10^−3^	6.53	0.0378
AC	6.250 × 10^−4^	1	6.250 × 10^−4^	0.97	0.3585
BC	2.500 × 10^−5^	1	2.500 × 10^−5^	0.039	0.8498
A^2^	3.727 × 10^−3^	1	3.727 × 10^−3^	5.76	0.0475
B^2^	5.084 × 10^−3^	1	5.084 × 10^−3^	7.86	0.0264
C^2^	0.088	1	0.088	136.32	<0.0001
Residual	4.530*E* − 003	7	6.471 × 10^−4^		
Lack of fit	1.450 × 10^−3^	3	4.833 × 10^−4^	0.63	0.6342
Pure error	3.080 × 10^−3^	4	7.700 × 10^−4^		
Cor total	0.23	16			

## Data Availability

The data used to support the findings of this study are included within the article.
